# Chronic posterior shoulder dislocation: Current evidence, treatment options, and a decision-making algorithm

**DOI:** 10.1016/j.jor.2026.05.015

**Published:** 2026-05-21

**Authors:** Raju Vaishya, Ayaz Saiyed, Abhishek Vaish, Rajesh Botchu, Patinharayil Gopinathan, Srinivas SB. Kambhampati, Filippo Migliorini

**Affiliations:** a Department of Orthopaedics, Indraprastha Apollo Hospitals, Sarita Vihar, New Delhi, 110076, India; b Department of Musculoskeletal Radiology, Royal Orthopedic Hospital, Birmingham, United Kingdom; c Department of Orthopaedics, GMC Ortho Foundation, Calicut, India; d Sri Dhaatri Orthopaedic, Maternity & Gynaecology Centre, Andhra Pradesh, India; e Department of Trauma and Reconstructive Surgery, University Hospital of Halle, Martin-Luther University Halle-Wittenberg, Halle (Saale), 06097, Germany; f Department of Life Sciences, Health, and Health Professions, Link Campus University of Rome, Via del Casale di San Pio V, Rome, 00165, Italy; g Department of Orthopaedic and Trauma Surgery, Eifelklinik St. Brigida, Kammerbruchstr. 8, Simmerath, 52152, Germany

**Keywords:** Chronic posterior shoulder dislocation, Locked posterior shoulder dislocation, Reverse Hill-Sachs lesion, McLaughlin procedure, Shoulder instability, Posterior

## Abstract

**Background and aims:**

Chronic posterior shoulder dislocation (CPSD), also known as locked or chronic posterior glenohumeral dislocation, is a rare but frequently missed injury, accounting for 2–5% of shoulder dislocations. It often results from trauma, seizures, or electrical shock, leading to reverse Hill-Sachs lesion (rHSL), posterior glenoid erosion, and soft-tissue contractures. Delayed diagnosis (50–79% initially overlooked) transforms acute cases into chronic ones (>3–6 weeks), complicating management and increasing risks of instability, osteoarthritis, and poor function. This narrative literature review aims to synthesise current evidence on the aetiology, diagnosis, imaging, treatment strategies, and outcomes of CPSD, and to propose a surgical decision-making algorithm to guide clinicians.

**Methods:**

A comprehensive search was conducted (up to March 13, 2026). Inclusion criteria included peer-reviewed articles (2006–2026) on adult patients with chronic PSD, focusing on interventions, clinical outcomes (pain, ROM, function, complications), and rehabilitation. Narrative synthesis was performed.

**Results:**

Aetiology is predominantly traumatic; pathoanatomy features rHSL (30–90% of cases) and posterior capsulolabral damage. Diagnosis relies on axillary radiographs, CT for bony defects, and MRI for soft tissues. Treatment depends on dislocation duration, rHSL size, glenoid integrity, and patient factors. A stepwise algorithm integrates reducibility, defect size, and associated pathologies.

**Conclusion:**

CPSD requires high clinical suspicion and advanced imaging for timely diagnosis. Joint-preserving reconstructions yield superior outcomes in moderate defects with early intervention; arthroplasty is preferred for extensive damage. The proposed algorithm provides structured guidance, though high-level evidence remains limited due to rarity and study heterogeneity. Future multicenter RCTs and standardised classifications are needed.

## Key Highlights

• Chronic PSD is often missed (50-79%); reverse Hill Sachs lesion (rHSL)is present in 30-90% cases and drives treatment.
• Treatment is often based on rHSL size: <25% conservative, 25-40% modified McLaughlin, >40-50% arthroplasty.
• Early surgery yields better outcomes; algorithm guides joint preservation vs. replacement.

## Graphical abstract

**Figure:**
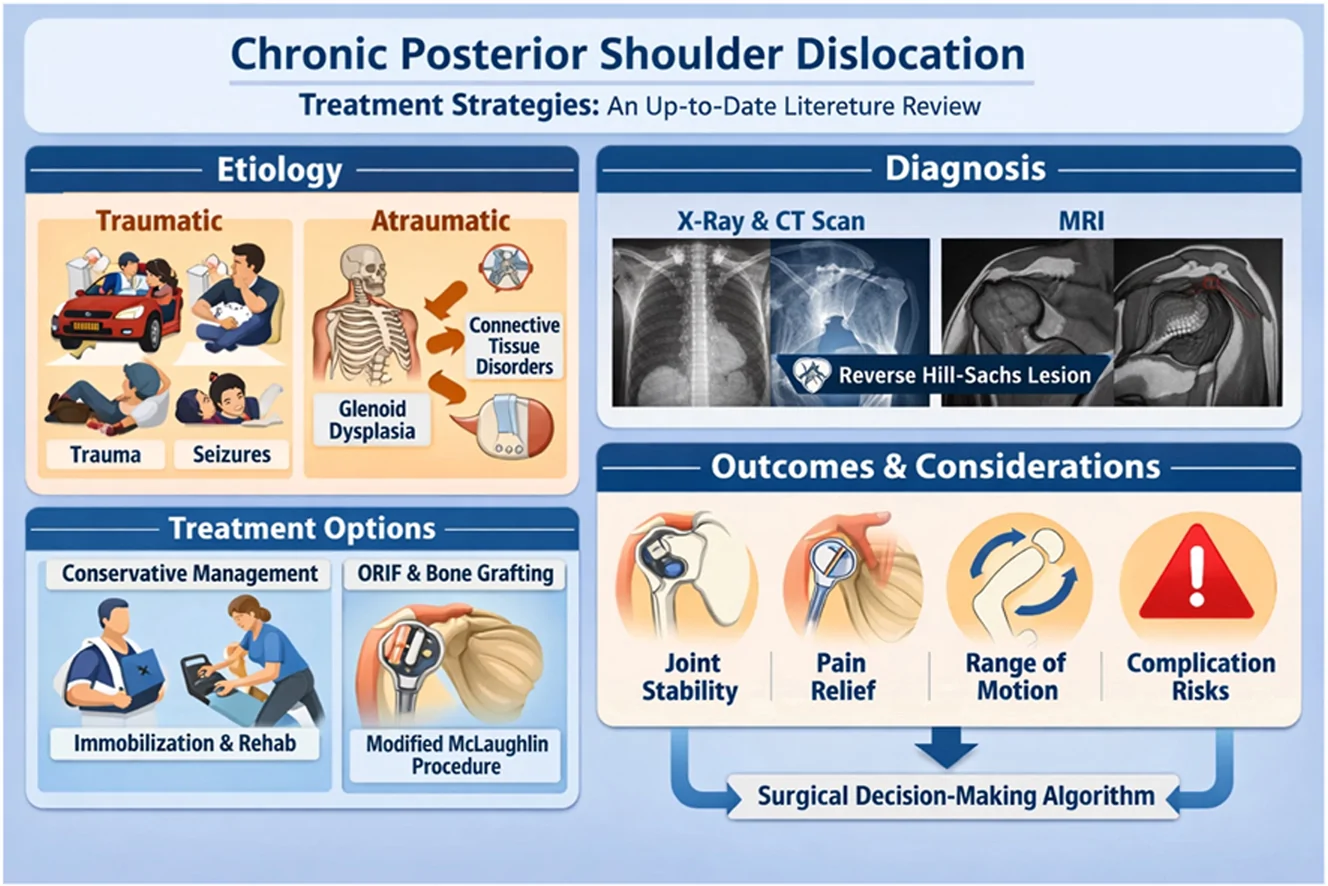

## 1 Introduction

The glenohumeral joint represents the most frequently dislocated joint in the human body. Posterior shoulder dislocation (PSD) is far less common than its anterior counterpart, yet it presents a major diagnostic difficulty. First documented by Sir Astley Cooper in 1839, this injury remains frequently missed. PSD accounts for 2–5% of all shoulder dislocations and typically results from high-energy trauma or powerful muscle contractions, such as those occurring during seizures or electrical injuries, though it can also develop gradually from repeated microtrauma that leads to chronic posterior instability.^1^^,^^2^ Atraumatic instances have been linked to connective tissue genetic conditions like Marfan syndrome or to bony anomalies such as glenoid hypoplasia and excessive retroversion.^3^

A PSD is classified as acute if the injury occurred within the past 3–6 weeks, and chronic beyond that timeframe. Nevertheless, certain recent investigations have questioned rigid time cutoffs, suggesting that closed reduction may still succeed in select chronic cases, thereby potentially broadening conservative management before resorting to surgery. Chronic posterior shoulder dislocation (CPSD) can manifest as recurrent episodes or as an unreduced/locked dislocation. Cases of long-standing unreduced PSD lasting more than 10 years have also been documented in the literature.^4^ Failure to recognise or properly reduce an acute PSD can quickly progress to a chronic locked state. Missed diagnoses commonly are due to misinterpretation of pain-restricted radiographs, restricted availability of advanced imaging like computed tomography (CT) in acute care settings, and a generally low clinical suspicion for PSD given its rarity relative to anterior dislocations. As a result, 50–79% of PSDs are initially overlooked, with many only identified after several weeks, by which point they qualify as chronic ^5^^,^^6^.

Recurrent posterior shoulder instability is less prevalent than anterior instability but is more frequently observed in specific populations, including certain young athletes and military personnel.^7^ The coexistence of soft-tissue and bony abnormalities plays a central role in the onset of posterior instability, and these can be effectively identified using magnetic resonance imaging (MRI) supplemented by CT scans.^8^ Any PSD persisting beyond six weeks is usually resistant to closed reduction and necessitates surgical management. A range of operative techniques has been reported for addressing CPSD, with the choice depending on the specific associated injuries present.^9^

Management of CPSD remains challenging because of the rarity of the condition and the variability of associated injuries. Treatment strategies range from conservative management and arthroscopic stabilisation to reconstructive procedures and shoulder arthroplasty, depending on the severity of structural damage. This review aims to synthesise the current evidence on chronic PSD, focusing on its management and suggesting a surgical decision-making algorithm for clinicians.

## 2 Methods

A comprehensive literature search was conducted across multiple databases, including PubMed, Scopus, and Google Scholar, limited to English-language articles published over the last 20 years to ensure relevance. The literature search was done on 13th March 2026, using keywords and phrases such as “Chronic posterior shoulder dislocation," “Posterior shoulder instability," “Humeral head reconstruction," “Modified McLaughlin procedure," “Shoulder arthroplasty," and “Rehabilitation protocols for shoulder dislocation," employing Boolean operators (AND, OR) to refine the results.

Inclusion criteria for the review encompassed: peer-reviewed original research articles, systematic reviews, meta-analyses, and clinical guidelines that involve adult patients (aged 18 years and older) diagnosed with chronic PSD. Studies discussing any form of treatment for chronic PSD, including surgical interventions like the modified McLaughlin procedure and arthroplasty, as well as non-surgical options such as physical therapy and rehabilitation protocols. The review focuses on studies reporting clinical outcomes, including pain relief, range of motion, functional recovery, and complications.

The exclusion criteria included editorials, letters to the editor, non-peer-reviewed articles, and studies involving pediatric patients or those with acute shoulder dislocations.

Data extraction was performed using a standardised form to collect relevant information from the included studies, including author(s), year of publication, study design, sample size, demographics, type of intervention, and key findings related to treatment outcomes. A narrative synthesis of the summarised findings was done.

## 3 Results and discussion

### 3.1 Aetiology

**Fig. 1:**
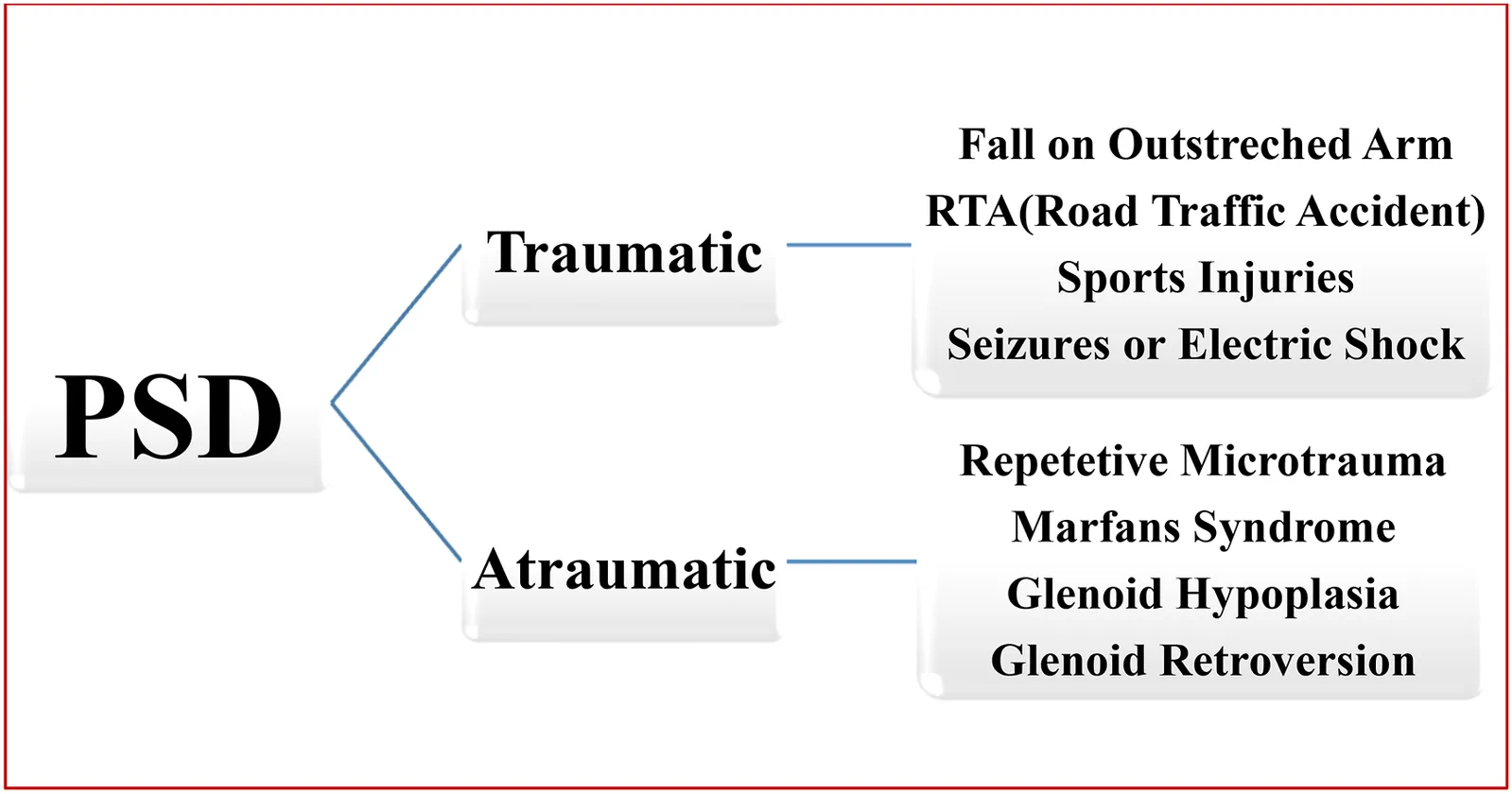
Aetiology of posterior shoulder dislocation (PSD).

The PSD can develop due to traumatic and atraumatic causes.^10^ In trauma, dislocation occurs when a force is applied along the arm's axis while the shoulder is flexed, adducted, and internally rotated, or due to violent muscle contractions. Whereas in atraumatic situations, dislocation occurs without significant injury, usually due to capsulolabral laxity or structural abnormalities (Fig. 1). Robinson et al. (2011) stated that most PSDs result from trauma (67%) and approximately 31% were caused by violent muscle contraction during seizures.^11^ Table 1 summarises the common causes of PSD.

**Table 1:** Common causes and mechanisms of posterior shoulder dislocation.

Cause	Mechanism	Clinical Scenario
Seizures	Violent involuntary contraction of internal rotator muscles (subscapularis, latissimus dorsi, pectoralis major)	Patients with epilepsy, metabolic disorders, or drug withdrawal
Electrical shock	Sudden simultaneous contraction of the shoulder girdle muscles	High-voltage electrical injuries
Trauma	Posteriorly directed force on an adducted, flexed, internally rotated arm	Falls, road traffic accidents, sports injuries
Direct blow to the anterior shoulder	Force pushes the humeral head posteriorly	Contact sports or blunt trauma
Iatrogenic	Improper manipulation or surgical complications	Following attempted reduction or surgery

### 3.2 Patho-anatomy

A CPSD leads to several distinctive structural alterations in the glenohumeral joint. A locked posterior shoulder dislocation (LPSD) manifests in three primary patterns: fracture-dislocation, impression fracture, and isolated dislocation without associated fracture. LPSD constitutes 2–4% of all shoulder dislocations, with an estimated annual incidence of 0.6 per 100,000 people.^12^

One of the most prevalent associated injuries is the reverse Hill–Sachs lesion (rHSL), an impaction fracture on the anteromedial aspect of the humeral head resulting from repeated impingement against the posterior glenoid rim during the dislocation event; this finding is present in 30–90% of cases.^12^ The majority of reported CPSD instances involve LPSDs. Additionally, damage to the posterior capsulolabral complex is common, with lesions affecting the posterior labrum and capsule that contribute to ongoing shoulder instability. Prolonged humeral head displacement in chronic cases can further cause progressive posterior glenoid bone loss through gradual erosion of the glenoid rim. Furthermore, long-standing dislocations often result in soft-tissue contractures**,** including capsular tightening and shortening of surrounding muscles, which further complicates reduction and surgical management.^13^ Accurate diagnosis of CPSD is critical and often requires advanced imaging techniques.

### 3.3 Imaging

Appropriate imaging is crucial for the diagnosis and management of CPSD. It not only helps diagnose the dislocation but also evaluates concurrent pathology. The findings from imaging studies directly influence treatment strategies.^14^ For instance, the size of the humeral head defect and the presence of associated injuries can dictate whether a conservative or surgical approach is more appropriate.

#### 3.3.1 Radiography

**Fig. 2:**
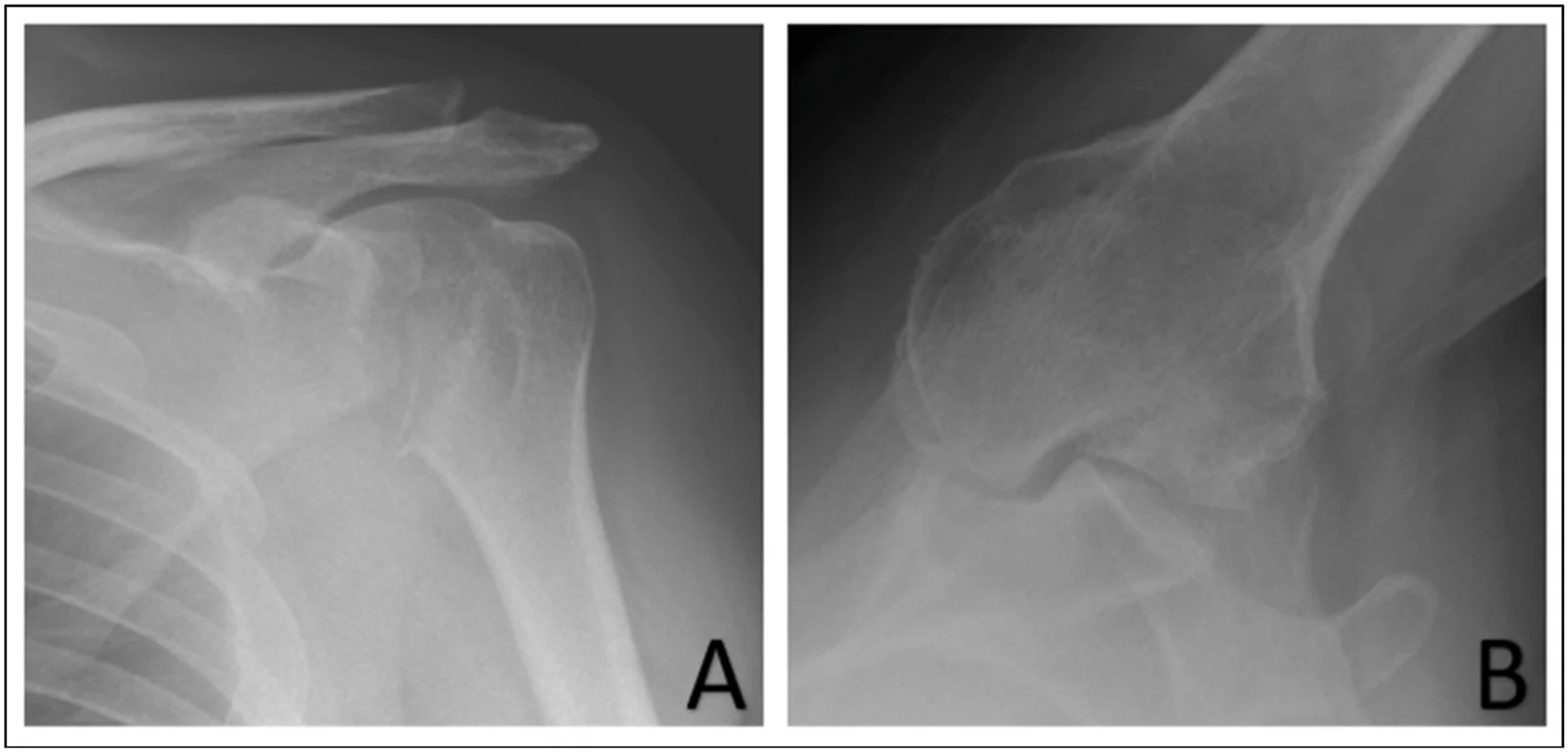
A) Anteroposterior (AP) and B) Axillary views of the left shoulder showing posterior dislocation of the shoulder with a humeral defect (reverse Hill-Sach's lesion).

**Fig. 3:**
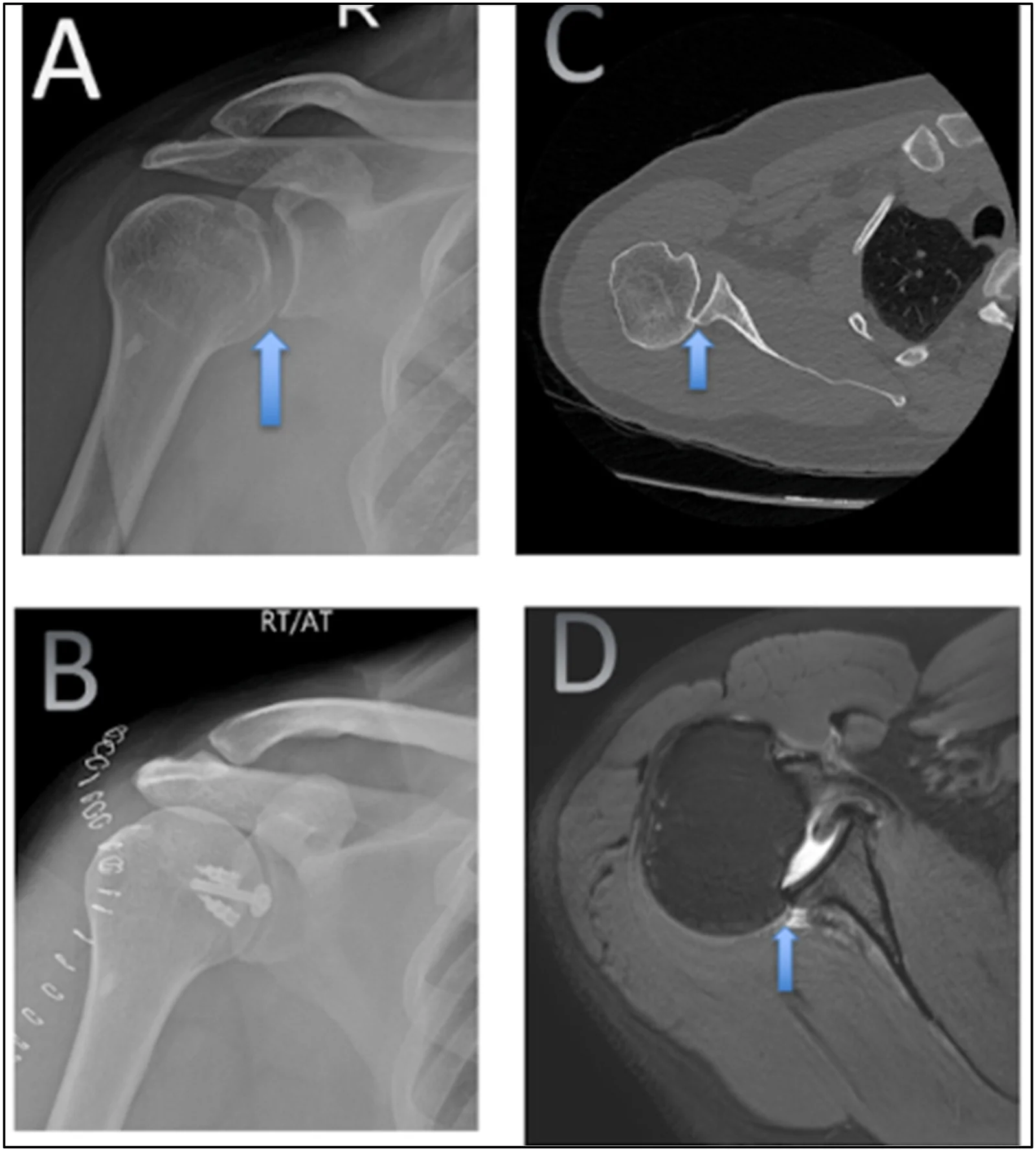
A) Anterior-posterior (AP) view showing dislocated shoulder with incongruous joint line (and light bulb sign); B) Postoperative radiograph showing internal fixation of the lesser tuberosity (Modified McLaughlin's procedure); C) CT image showing posterior dislocation with a humeral head defect; D) MRI (PDFS – proton density fat suppressed axial) image showing posterior dislocation with a humeral head defect (reverse Hill-Sachs lesion).

Plain radiographs are essential for initial assessment, but may not always reveal the extent of the injury. Two views are obligatory: anteroposterior (AP) and axial (Fig. 2). If pain precludes an axial x-ray because of limited abduction, a ‘scapular- Y’ view is valuable. Several characteristic radiographic signs may suggest PSD, including the light bulb sign (Fig. 3A), rim sign, and trough line sign (Table 2), but these are not 100% diagnostic, and dislocation is thus difficult to detect.^15^

**Table 2:** Clinical and radiological signs of posterior shoulder dislocation.

Feature	Description
Arm position	Adduction and internal rotation
External rotation	Severely limited or impossible
Shoulder contour	Flattened anterior shoulder with posterior fullness
Coracoid prominence	More prominent anteriorly
Light bulb sign (X-ray)	Symmetrical, rounded appearance of the humeral head due to internal rotation
Rim sign	Widened glenohumeral joint space (>6 mm)
Trough line sign	Vertical line on the medial humeral head indicating an impaction fracture
Axillary view	Shows posterior displacement of the humeral head

#### 3.3.2 MRI and CT

These imaging techniques offer detailed visualisation of both soft tissue and osseous structures, aiding in the detection of associated injuries and the severity of humeral defects. CT is particularly valuable for evaluating bony defects of the glenoid and humeral head (Fig. 3C), making it indispensable for preoperative planning. MRI, especially when performed with contrast, is highly effective in assessing soft-tissue injuries (Fig. 3D), including labral and rotator cuff lesions, and in identifying incarceration of the long head of the biceps tendon in irreducible dislocations. In contrast to anterior shoulder dislocations, which typically involve soft tissue injuries of the anterior labrum and capsule, posterior dislocations are more commonly associated with bony lesions such as the anterior humeral head impression fracture (reverse Hill–Sachs lesion) and the McLaughlin lesion (l'encoche de Malgaigne).^16^^,^^17^

### 3.4 Treatment

The management of PSD is complex and ranges from conservative observation to total shoulder arthroplasty (TSA). Key factors influencing treatment decisions include the size of the reverse Hill–Sach's lesion (rHSL), duration of dislocation, condition of the glenoid, and the patient's age and overall health.^18^

#### 3.4.1 Non-operative/conservative treatment

Conservative management may be appropriate in acute cases with a stable shoulder and small bony defects (<25% of the articular surface) following successful closed reduction. The shoulder is typically immobilised in internal rotation or a neutral position for a brief period. However, outcomes with non-operative treatment are generally inferior to surgical management, particularly in chronic presentations.^19^

#### 3.4.2 Operative/surgical treatment

Surgical intervention should be considered in patients with persistent pain, instability, or functional impairment despite 3–6 months of conservative therapy.^20^ A 2017 prospective multicentre non-randomised study by the French Society of Arthroscopy demonstrated superior outcomes with surgical treatment compared to non-operative care.^21^

Surgery is indicated for symptomatic posterior labral tears, unidirectional posterior instability with capsular insufficiency, and selected cases of multidirectional instability with predominant posterior symptoms. Both open and arthroscopic approaches are effective, with arthroscopy offering the advantage of addressing concomitant intra-articular lesions in the same setting.^22^ Arthroscopic posterior stabilisation has shown good to excellent results, with high satisfaction and low recurrence rates. In a large prospective series, Bradley et al. reported 94% patient satisfaction following labral repair with or without capsular plication.^23^ Similarly, Wooten et al. noted 92% satisfaction, a 67% return-to-play rate without restrictions, and an 8% failure rate in young athletes.^24^

The choice of surgical technique is largely guided by defect size. Conservative treatment is typically sufficient for defects <25% in a stable joint. Reconstruction of the articular surface is recommended for defects between 25% and 40%, while defects exceeding 40% generally require prosthetic replacement.^25^ In chronic posterior shoulder dislocation (CPSD), management is primarily dictated by the size of the rHSL or humeral head defect (Table 3). Earlier surgical intervention is associated with better functional outcomes, including higher Constant scores and improved external rotation, whereas delays beyond 6 months are linked to worse results and an increased risk of osteoarthritis (OA). Several surgical techniques have been described for addressing humeral head defects.

**Table 3:** Treatment strategy based on the size of reverse Hill-Sachs lesion.

Size of Humeral Head Defect	Recommended Treatment
<20–25%	Closed reduction or open reduction with soft tissue repair
25–40%	McLaughlin procedure or modified McLaughlin (lesser tuberosity transfer)
30–50%	Bone graft reconstruction or osteochondral graft
>40–50%	Shoulder arthroplasty (hemiarthroplasty or total shoulder arthroplasty)

For defects involving approximately 25–40% of the humeral head, reconstructive procedures such as the McLaughlin procedure or the modified McLaughlin technique are commonly performed.^26^ In cases where the defect ranges from about 30 to 50%, bone graft reconstruction or osteochondral grafting may be required to restore the humeral head contour and maintain joint stability. When the defect exceeds 40–50% of the humeral head surface, joint-preserving procedures become less effective, and shoulder arthroplasty, including hemiarthroplasty or total shoulder arthroplasty (TSA), is usually recommended^27^ (Table 4).

**Table 4:** Common surgical options for chronic posterior shoulder dislocation.

Procedure	Indications	Advantages	Limitations
McLaughlin procedure	Reverse Hill-Sachs defect 20–40%	Prevents the engagement of the defect	Limited for large defects
Modified McLaughlin	Moderate defects with chronic dislocation	Better structural filling of the defect	Technically demanding
Bone graft reconstruction	Large defects in younger patients	Preserves the native joint	Risk of graft resorption
Rotational osteotomy	Historically used for moderate defects	Restores alignment	Rarely used today
Shoulder arthroplasty	Massive defects (>40–50%) or arthritis	Reliable pain relief	Limited longevity in young patients

##### 3.4.2.1 McLaughlin (and modified) procedures

The McLaughlin's procedure, first described by Robert McLaughlin in 1952, is used for posterior dislocations with moderate humeral head defects. The surgery involves the transfer of the subscapularis tendon into the defect created by an rHSL, and it is commonly performed arthroscopically. The technique has the advantage of being relatively simple; however, it may not fill larger defects of the humeral head fully. The modified McLaughlin's procedure, later described by Richard J. Hawkins, Charles Neer and colleagues, involves transfer of the lesser tuberosity along with the attached subscapularis tendon into the humeral head defect (Fig. 3B). This modification is commonly used for moderate defects ranging from 25 to 40% and provides stronger structural filling of the defect with a more anatomical reconstruction.

Although the modified technique is more technically challenging, it generally provides greater joint stability and a reduced risk of recurrence, with several studies reporting better functional outcomes than the original procedure. Arthroscopic modified McLaughlin techniques, particularly when combined with posterior labral repair, are especially useful in addressing associated intra-articular pathology.

A systematic review^28^ suggested that the arthroscopic McLaughlin technique may be preferable in acute irreducible posterior dislocations with minimal bone loss (<20%), even though both approaches yield comparable functional outcomes. However, Besnard et al. (2019) noted a potential drawback of the arthroscopic technique, namely a theoretical reduction in internal rotation.^29^ In contrast, Georgios Paparoidamis et al. (2020)^30^ reported that the modified technique achieves superior functional outcomes compared to arthroscopic management in cases of CPSD with bone loss of less than 40%. A comparative overview of these techniques is presented in Table 5.

**Table 5:** Comparison of McLaughlin's and modified McLaughlin's procedures.

Feature	McLaughlin Procedure	Modified McLaughlin Procedure
First described	McLaughlin (1952)	Hawkins et al. modification
Principle	Transfer of the subscapularis tendon into the humeral head defect	Transfer of the lesser tuberosity with attached subscapularis
Indications	Reverse Hill-Sachs defect 20–40%	Moderate defects (25–40%)
Stability	Good stability in moderate defects	Provides stronger structural filling
Advantages	Relatively simple technique	More anatomical reconstruction
Limitations	May not fill larger defects fully	Technically more demanding
Clinical outcomes	Generally good functional recovery	Often superior stability and reduced recurrence

##### 3.4.2.2 Bone grafting

Many cases of CPSD with large rHSL or posterior glenoid bone loss necessitate reconstruction using bone grafting. The primary goal is to restore the normal contour of the glenohumeral joint, thereby reducing recurrent instability and minimizing the risk of long-term OA.^31^ In younger patients with humeral head defects exceeding 45%, open reduction combined with stabilisation and bone grafting is generally considered the preferred treatment option.^32^

Various techniques have been described for addressing these defects (Table 6), including the use of allografts (such as femoral head, humeral head, or fresh-frozen grafts), autografts (e.g., coracoid process or iliac crest), and bone graft substitutes.^33^ Allografts offer the advantage of avoiding donor site morbidity associated with iliac crest harvesting; however, they carry risks such as infection, possible viral transmission, non-union, immune reactions, and the need for specialized resources.^34^ Miniaci and Gish^35^ reported satisfactory outcomes using fresh-frozen, size-matched osteochondral allografts in 18 cases with defects >25%, achieving a mean Constant score of 78.5 at an average follow-up of 50 months. Similarly, Diklic et al.^36^ observed a mean Constant score of 86.8 at 54 months following reconstruction with femoral allografts fixed using cannulated screws. Gerber and Lambert^37^ also demonstrated favorable outcomes, with a mean Constant score of 82 in four patients treated with femoral allografts.

**Table 6:** Types of Bone Grafting used in the management of Chronic Posterior Shoulder Dislocation.

Graft type	Indication/Use	Advantages
Femoral head allograft	Large reverse Hill–Sachs lesions (>25–45%)	Restores articular congruity; no donor site morbidity; good size match
Humeral head allograft	Segmental humeral head defects	Anatomical reconstruction; preserves the joint
Fresh-frozen osteochondral allograft	Large osteochondral defects	Better cartilage restoration; joint preservation in young patients.
Iliac crest autograft	Large humeral head defects or glenoid reconstruction	Readily available; no disease transmission; good incorporation
Coracoid process autograft	Selected humeral head defects/instability	Local graft; adds stability
Distal clavicle autograft	Posterior glenoid reconstruction	Local graft; avoids second surgical site
Distal tibial allograft	Posterior glenoid bone loss	Good contour match; restores articular surface

Management of posterior glenoid bone loss in CPSD remains particularly challenging. Several innovative reconstructive techniques have been described, including iliac crest bone block, distal tibial allograft, glenoid allograft, pedunculated acromial graft, and distal clavicular autograft.^38^ Although these posterior bone block procedures have shown encouraging clinical results, they are technically demanding and associated with complication rates of up to 36%, including persistent instability and the development of OA.^39^

In a multicenter study, Clavert et al.^40^ reported significant improvements in Constant and Walch-Duplay scores, indicating good clinical outcomes. However, recurrent instability was noted in 12% of cases, and 18% of patients continued to experience shoulder pain after surgery. Additionally, Schwartz et al.^41^ found that 36.8% of shoulders required revision surgery following bone block procedures, despite the absence of recurrent instability.

##### 3.4.2.3 Osteotomies

Patients with significant glenoid dysplasia (retroversion >15°) or posterior glenoid bone loss have a higher likelihood of failure following soft tissue repair due to compromised bony support.^42^ In select and uncommon situations, excessive glenoid retroversion may be corrected with a posterior glenoid osteotomy, most often in revision cases. Graichen et al.^43^ reported good to excellent outcomes in 81% of 32 patients undergoing this procedure, with the most favorable results seen in those with atraumatic instability. However, the development of postoperative OA in 25% of cases highlights that this option should be reserved for carefully selected patients.

Given the limitations of articular cartilage elevation techniques, particularly in chronic cases (>6 months), graft incorporation may be problematic due to compromised vascularity of the humeral head, potentially leading to collapse and recurrent instability. Humeral derotational osteotomy has also been described as a treatment option for CPSD.^44^

##### 3.4.2.4 Arthroplasty

Shoulder arthroplasty is indicated in complex posterior fracture-dislocations, especially when open reduction has failed, when more than 50% of the humeral head is involved, or when secondary OA has developed. Hemiarthroplasty is generally recommended in patients over 65 years with acute three- or four-part humeral head fractures because of the high risk of devascularization.^45^ Hawkins et al.^46^ suggested prosthetic replacement in cases with dislocations lasting longer than six months and defects exceeding 45% of the humeral head. However, more recent evidence indicates that functional outcomes following shoulder arthroplasty may be inferior to those achieved with successful open reduction and internal fixation (ORIF).

### 3.5 Management algorithm

**Fig. 4:**
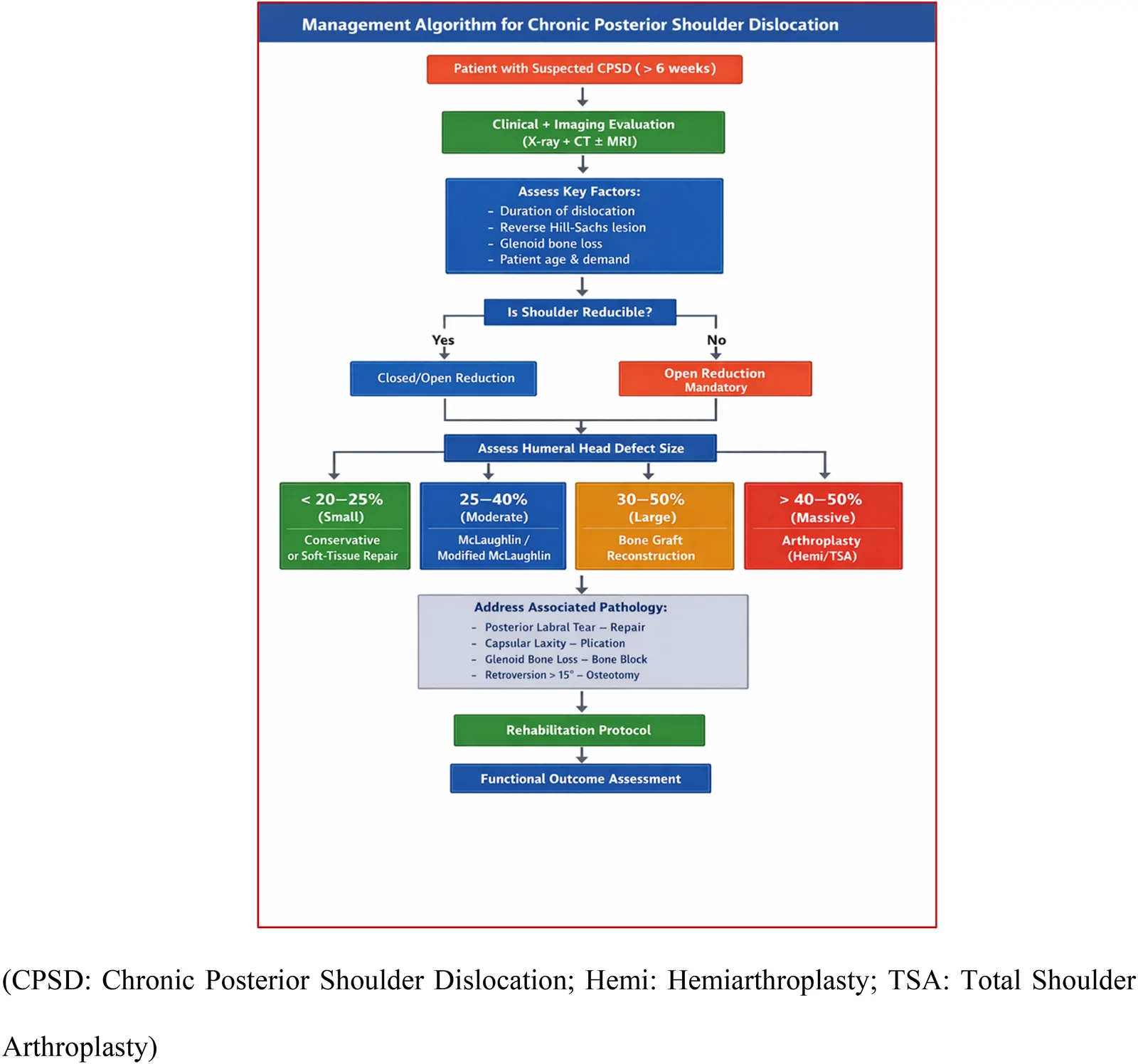
Management algorithm for chronic posterior shoulder Dislocation (CPSD: Chronic posterior shoulder dislocation; hemi: Hemiarthroplasty; TSA: Total shoulder arthroplasty).

Based on the review's core findings, we propose a management algorithm that provides a structured, stepwise approach to the management of CPSD, emphasising individualised decision-making based on key pathological and patient-specific factors (Fig. 4). Following clinical and imaging evaluation, the primary determinant is the joint's reducibility, which guides the need for open versus closed reduction. The size of the rHSL plays a pivotal role in selecting treatment: smaller defects may be managed conservatively or with soft tissue procedures, while moderate defects typically require reconstructive techniques such as the McLaughlin's or modified McLaughlin's procedure. Larger defects necessitate bone grafting, and massive defects are best treated with arthroplasty. Importantly, the algorithm integrates the management of associated pathologies, such as labral tears, capsular laxity, and glenoid bone loss, to optimise stability and outcomes. Rehabilitation and functional assessment form the final steps, ensuring restoration of shoulder function and long-term success.

### 3.6 Rehabilitation

A structured and well-targeted rehabilitation program is essential for maximising recovery and enabling patients to return to full activity.^47^ Rehabilitation is generally divided into four phases: (I) protection, (II) active range of motion (ROM) and muscle endurance, (III) strengthening and power development, and (IV) return to sport (Table 7).

1. *Protection phase* – During this phase, the shoulder is immobilised in a sling with an abduction pillow to maintain the glenohumeral joint in the scapular plane and reduce stress on the repair. In cases with posterior defects, a thoracic abduction splint may be used for up to 6 weeks. Cryotherapy is an important adjunct for alleviating pain, reducing muscle spasm, and controlling swelling.^48^
2. *Active ROM and muscle endurance* – Rehabilitation progresses to active-assisted range of motion (AAROM), followed by active range of motion (AROM), typically around 5–6 weeks postoperatively. As the repair is still in the early healing phase, exercises are carefully selected to avoid excessive strain. Passive external rotation may be introduced within 0–12 weeks, depending on the surgical technique and individual patient factors.^49^
3. *Muscular strengthening and power* – Strengthening exercises are usually initiated at approximately 12–13 weeks postoperatively. The aim is to improve shoulder dynamic stability while ensuring controlled loading, particularly in posterior directions, to restore optimal upper limb function. Progressive resistance exercises, including bench press and push-ups, are typically incorporated between 13 and 15 weeks.^50^
4. *Return to sport* – This phase involves a gradual reintroduction to sport-specific activities, guided by the patient's symptoms and joint tolerance. Rest days should be utilised for maintenance exercises focusing on flexibility, cardiovascular fitness, and upper-limb and core strengthening. Return to sports or high-demand activities is generally advised around 21–22 weeks after surgery.^51^

**Table 7:** Key rehabilitation phases.

Phase	Duration	Focus
Immobilization	4-6 weeks	Sling or splint use; early ROM for elbow, wrist, and hand.
Early Range of Motion (ROM)	0-12 weeks	Gradual introduction of passive and active-assisted ROM.
Strengthening	13-15 weeks	Scapular and shoulder muscle strengthening; resistance exercises.
Return to Sport	∼21-22 weeks	Functional training and sport-specific activities.

### 3.7 Complications

Potential complications of surgical procedures for CPSD include recurrent instability, avascular necrosis (AVN) of the humeral head, OA, and postoperative stiffness.^52^ The possible causes and the clinical impact of these complications are summarised in Table 8. Early diagnosis significantly reduces complication rates.

**Table 8:** Complications reported after treatment of chronic posterior shoulder dislocation.

Complication	Possible Cause	Clinical Impact
Recurrent instability	Inadequate defect reconstruction	Persistent shoulder instability
Avascular necrosis of the humeral head	Disruption of the blood supply	Collapse of the humeral head
Post-traumatic osteoarthritis (OA)	Chronic cartilage damage	Progressive pain and stiffness
Shoulder stiffness	Prolonged immobilization or capsular contracture	Reduced range of motion
Graft failure/resorption	Poor integration of the bone graft	Loss of humeral head contour
Implant complications	Arthroplasty-related issues	Revision surgery may be required

### 3.8 Limitations

This review has several limitations that should be acknowledged. First, the available literature on CPSD is limited by the rarity of the condition, resulting in a predominance of small case series and retrospective studies with heterogeneous patient populations, surgical techniques, and outcome measures. The absence of high-quality randomised controlled trials and standardised treatment protocols restricts the ability to draw definitive conclusions or establish universally accepted guidelines. Additionally, variability in the definition of “chronicity,” differences in imaging assessment, and inconsistent reporting of functional outcomes and complications further limit comparability across studies. Publication bias toward positive surgical outcomes may also influence the overall interpretation of treatment efficacy. Furthermore, there is significant variability in rehabilitation protocols across institutions, particularly regarding the timing of exercises and functional milestones.

### 3.9 Future direction

Future research should focus on multicenter prospective studies and randomised controlled trials (RCTs) to generate higher-level evidence and enable standardised comparisons between different surgical techniques. Development of a universally accepted classification system incorporating defect size,^52^ chronicity, and associated soft tissue pathology would improve treatment stratification. Long-term follow-up studies are needed to better evaluate functional outcomes, complication rates, and the durability of joint-preserving procedures versus arthroplasty. Furthermore, advances in imaging, patient-specific surgical planning, and biologic augmentation techniques may enhance treatment outcomes. Establishing standardised rehabilitation protocols and reporting guidelines will also be essential to optimise patient recovery and allow meaningful comparison across future studies.

## 4 Conclusion

Chronic posterior shoulder dislocation (CPSD) is a rare and frequently missed injury. A high index of suspicion, careful clinical examination, and appropriate imaging are essential for early diagnosis. Treatment strategies depend largely on the size of the reverse Hill-Sachs lesion (rHSL) and duration of dislocation. While reconstructive procedures can restore joint stability in moderate defects, shoulder arthroplasty remains the treatment of choice for extensive humeral head damage. The outcomes of surgical procedures depend mainly on the duration of dislocation, the size of the humeral head defect, and the type of surgical intervention. Patients treated early with reconstructive procedures generally achieve good functional outcomes, whereas delayed treatment often requires arthroplasty with variable results. This review article provides a clinical decision-making algorithm for the management of CPSD.

## Consent to participate

Not applicable.

## Consent to publish

Not applicable.

## Guardian/patient's consent

Not applicable.

## Author's contribution

Conceptualisation: RV, AS, AV; Literature Search & Data Analysis: RV, AS, AV; Manuscript writing, editing and final approval: RV, AS, AV, RB, PG, SBSK, FP, FM.

## Ethical approval

This study complies with ethical standards.

## Registration and protocol

The present study was not registered.

## Human ethics and consent to participate declarations

Not applicable.

## Use of AI

We used ChatGPT (4.0) to review the article and improve its English grammar and readability. However, the final manuscript has been read by all the authors, and we accept the full responsibility for its contents.

## Funding

No funding was received.

The authors received no financial support for the research, authorship, and/or publication of this article.

## Conflict of interest

The authors declare that they have no competing interests for this article.

## Data Availability

This review is based on the published literature, which is already publicly available.
